# Effect of internet use and electronic game-play on academic performance of Australian children

**DOI:** 10.1038/s41598-020-78916-9

**Published:** 2020-12-10

**Authors:** Md Irteja Islam, Raaj Kishore Biswas, Rasheda Khanam

**Affiliations:** 1 Centre for Health Research and School of Commerce, University of Southern Queensland, Workstation 15, Room T450, Block T, Toowoomba, QLD 4350 Australia; 2 Maternal and Child Health Division, International Centre for Diarrhoeal Disease Research, Bangladesh (icddr,b), Mohakhali, Dhaka, 1212 Bangladesh; 3 Transport and Road Safety (TARS) Research Centre, School of Aviation, University of New South Wales, Sydney, NSW 2052 Australia

**Keywords:** Psychology, Human behaviour, Risk factors

## Abstract

This study examined the association of internet use, and electronic game-play with academic performance respectively on weekdays and weekends in Australian children. It also assessed whether addiction tendency to internet and game-play is associated with academic performance. Overall, 1704 children of 11–17-year-olds from young minds matter (YMM), a cross-sectional nationwide survey, were analysed. The generalized linear regression models adjusted for survey weights were applied to investigate the association between internet use, and electronic-gaming with academic performance (measured by NAPLAN–National standard score). About 70% of the sample spent > 2 h/day using the internet and nearly 30% played electronic-games for > 2 h/day. Internet users during weekdays (> 4 h/day) were less likely to get higher scores in reading and numeracy, and internet use on weekends (> 2–4 h/day) was positively associated with academic performance. In contrast, 16% of electronic gamers were more likely to get better reading scores on weekdays compared to those who did not. Addiction tendency to internet and electronic-gaming is found to be adversely associated with academic achievement. Further, results indicated the need for parental monitoring and/or self-regulation to limit the timing and duration of internet use/electronic-gaming to overcome the detrimental effects of internet use and electronic game-play on academic achievement.

## Introduction

Over the past two decades, with the proliferation of high-tech devices (e.g. Smartphone, tablets and computers), both the internet and electronic games have become increasingly popular with people of all ages, but particularly with children and adolescents^1–3^. Recent estimates have shown that one in three under-18-year-olds across the world uses the Internet, and 75% of adolescents play electronic games daily in developed countries^4–6^. Studies in the United States reported that adolescents are occupied with over 11 h a day with modern electronic media such as computer/Internet and electronic games, which is more than they spend in school or with friends^7,8^. In Australia, it is reported that about 98% of children aged 15–17 years are among Internet users and 98% of adolescents play electronic games, which is significantly higher than the USA and Europe^9–12^.

In recent times, the Internet and electronic games have been regarded as important, not just for better results at school, but also for self-expression, sociability, creativity and entertainment for children and adolescents^13,14^. For instance, 88% of 12–17 year-olds in the USA considered the Internet as a useful mechanism for making progress in school^15^, and similarly, electronic gaming in children and adolescents may assist in developing skills such as decision-making, smart-thinking and coordination^3,15^.

On the other hand, evidence points to the fact that the use of the Internet and electronic games is found to have detrimental effects such as reduced sleeping time, behavioural problems (e.g. low self-esteem, anxiety, depression), attention problems and poor academic performance in adolescents^1,5,12,16^. In addition, excessive Internet usage and increased electronic gaming are found to be addictive and may cause serious functional impairment in the daily life of children and adolescents^1,12,13,16^. For example, the AU Kids Online survey^17^ reported that 50% of Australian children were more likely to experience behavioural problems associated with Internet use compared to children from 25 European countries (29%) surveyed in the EU Kids Online study^18^, which is alarming^12^. These mixed results require an urgent need of understanding the effect of the Internet use and electronic gaming on the development of children and adolescents, particularly on their academic performance.

Despite many international studies and a smaller number in Australia^12^, several systematic limitations remain in the existing literature, particularly regarding the association of academic performance with the use of Internet and electronic games in children and adolescents^13,16,19^. First, the majority of the earlier studies have either relied on school grades or children’s self assessments—which contain an innate subjectivity by the assessor; and have not considered the standardized tests of academic performance^16,20–22^. Second, most previous studies have tested the hypothesis in the school-based settings instead of canvassing the whole community, and cannot therefore adjust for sociodemographic confounders^9,16^. Third, most studies have been typically limited to smaller sample sizes, which might have reduced the reliability of the results^9,16,23^.

By considering these issues, this study aimed to investigate the association of internet usage and electronic gaming on a standardized test of academic performance—NAPLAN (The National Assessment Program—Literacy and Numeracy) among Australian adolescents aged 11–17 years using nationally representative data from the Second Australian Child and Adolescent Survey of Mental Health and Wellbeing—Young Minds Matter (YMM). It is hypothesized that the findings of this study will provide a population-wide, contextual view of excessive Internet use and electronic games played separately on weekdays and weekends by Australian adolescents, which may be beneficial for evidence-based policies.

## Results

### Subject demographics

Respondents who attended gave NAPLAN in 2008 (N = 4) and 2009 (N = 29) were removed from the sample due to smaller sample size, as later years (2010–2015) had over 100 samples yearly. The NAPLAN scores from 2008 might not align with a survey conducted in 2013. Further missing cases were deleted with the assumption that data were missing at random for unbiased estimates, which is common for large-scale surveys^24^. From the initial survey of 2967 samples, 1704 adolescents were sampled for this study.

The sample characteristics were displayed in Table 1. For example, distribution of daily average internet use was checked, showing that over 50% of the sampled adolescents spent 2–4 h on internet (Table 1). Although all respondents in the survey used internet, nearly 21% of them did not play any electronic games in a day and almost one in every three (33%) adolescents played electronic games beyond the recommended time of 2 h per day. Girls had more addictive tendency to internet/game-play in compare to boys.

**Table 1:** Sample characteristics.

Variables	Girls	Boys	Total
	N (%)	N (%)	N (%)
Age*			
	15.42 (1.38)	15.35 (1.38)	15.38 (1.38)
**Household income**			
Low	163 (20)	157 (18.3)	320 (19.2)
Medium	377 (46.3)	404 (47.2)	781 (46.7)
High	275 (33.7)	295 (34.5)	570 (34.1)
**Primary carer's highest level of education**			
Bachelor	268 (32.9)	287 (33.5)	555 (33.2)
Diploma	304 (37.3)	317 (37)	621 (37.2)
Year-10_11	243 (29.8)	252 (29.4)	495 (29.6)
**Family structure**			
Original	517 (63.4)	576 (67.3)	1093 (65.4)
Step	42 (5.2)	57 (6.7)	99 (5.9)
Blended	62 (7.6)	54 (6.3)	116 (6.9)
Sole-parent/care	183 (22.5)	160 (18.7)	343 (20.5)
Other	11 (1.3)	9 (1.1)	20 (1.2)
**Remoteness**			
Major city	540 (66.3)	529 (61.8)	1069 (64)
Inner regional	209 (25.6)	242 (28.3)	451 (27)
Outer regional	60 (7.4)	73 (8.5)	133 (8)
Remote	6 (0.7)	12 (1.4)	18 (1.1)
**Primary carer's likelihood of serious mental illness (K6 score)**			
Likely	23 (2.8)	21 (2.5)	44 (2.6)
Not likely	792 (97.2)	835 (97.5)	1627 (97.4)
**Primary carer's smoking status**			
No	684 (83.9)	702 (82)	1386 (82.9)
Yes	131 (16.1)	154 (18)	285 (17.1)
**Risk of alcohol related harm by the primary carer**			
Risky	212 (26)	246 (28.7)	458 (27.4)
None	603 (74)	610 (71.3)	1213 (72.6)
**Daily internet use**			
≤ 2 h	149 (18.3)	203 (23.7)	352 (21.1)
2–4 h	448 (55)	416 (48.6)	864 (51.7)
> 4 h	218 (26.7)	237 (27.7)	455 (27.2)
**Daily game-play**			
0 h	276 (33.9)	69 (8.1)	345 (20.6)
1–2 h	403 (49.4)	375 (43.8)	778 (46.6)
> 2 h	136 (16.7)	412 (48.1)	548 (32.8)
**Addiction tendency to internet and/or game-play**			
Yes	143 (17.5)	131 (15.3)	274 (16.4)
No	672 (82.5)	725 (84.7)	1397 (83.6)

*Mean and SD.

The mean scores for the three NAPLAN tests scores (reading, writing and numeracy) ranged from 520 to 600. A gradual decline in average NAPLAN tests scores (reading, writing and numeracy) scores were observed for internet use over 4 h during weekdays, and over 3 h during weekends (Table 2). Table 2 also shows that adolescents who played no electronic games at all have better scores in writing compared to those who play electronic games. Moreover, Table 2 shows no particular pattern between time spent on gaming and NAPLAN reading and numeracy scores. Among the survey samples, 308 adolescents were below the national standard average.

**Table 2:** Average NAPLAN scores for reading, writing and numeracy across average daily internet use and average daily game-play.

In hours	Weekdays [Mean (SD)]				Weekends [Mean (SD)]			
	Sample size	Reading	Writing	Numeracy	Sample size	Reading	Writing	Numeracy
**Average daily internet use**								
1	167	574.48 (71.6)	541.78 (104.63)	592.16 (84.74)	140	561.54 (69.55)	527.33 (109.13)	573.28 (79.20)
2	406	594.63 (75.64)	569.75 (97.48)	598.82 (73.02)	325	580.04 (68.73)	557.43 (85.58)	589.34 (70.14)
3	500	582.11 (65.44)	563.35 (81.79)	586.45 (67.02)	460	594.42 (68.33)	574.23 (81.55)	597.18 (69.90)
4	291	594.63 (65.45)	570.61 (88.31)	593.02 (70.96)	331	586.87 (71.66)	565.98 (92.34)	591.86 (75.33)
5	119	576.41 (63.18)	565.23 (84.14)	581.25 (76.59)	191	588.36 (ss68.35)	569.43 (94.09)	590.03 (73.04)
6	81	581.21 (69.6)	555.23 (93.03)	585.36 (64.48)	119	585.26 (62.86)	560.08 (86.42)	587.22 (74.73)
7	140	571.07 (62.65)	546.63 (84.00)	574.71 (73.70)	138	581.42 (63.41)	553.60 (93.60)	578.77 (65.14)
**Average daily game-play**								
0	355	586.84 (67.43)	578.45 (84.22)	583.76 (68.63)	355	586.84 (67.43)	578.45 (84.22)	583.76 (68.63)
1	608	593.92 (67.61)	576.48 (82.98)	602.54 (74.76)	394	590.08 (67.46)	574.23 (82.66)	593.60 (69.92)
2	398	580.86 (73.11)	551.91 (99.13)	583.02 (71.94)	377	581.67 (75.99)	563.46 (98.64)	591.13 (79.62)
3	195	577.30 (61.29)	542.34 (90.48)	586.41 (67.23)	295	586.07 (66.18)	551.02 (80.46)	592.06 (73.02)
4	69	562.31 (69.17)	516.22 (70.88)	566.93 (64.47)	134	580.04 (66.45)	540.57(107.64)	593.29 (72.75)
5	42	583.09 (75.94)	545.22 (102.6)	584.92 (81.16)	67	582.21 (62.33)	529.96 (95.37)	581.15 (64.58)
6	7	568.60 (53.89)	520.10 (35.58)	562.44 (45.88)	37	598.60 (62.53)	561.82 (67.99)	610.53 (73.08)
7	30	553.73 (66.11)	495.29 (105.97)	575.28 (78.90)	45	559.79 (63.89)	510.79 (97.96)	559.96 (52.86)

### Internet use and academic performance

Our results show that internet (non-academic use) use during weekdays, especially more than 4 h, is negatively associated with academic performance (Table 3). For internet use during weekdays, all three models showed a significant negative association between time spent on internet and NAPLAN reading and numeracy scores. For example, in Model 1, adolescents who spent over 4 h on internet during weekdays are 15% and 17% less likely to get higher reading and numeracy scores respectively compared to those who spend less than 2 h. Similar results were found in Model 2 and 3 (Table 3), when we adjusted other confounders. The variable addiction tendency to internet was found to be negatively associated with NAPLAN results. The adolescents who had internet addiction were 17% less and 14% less likely to score higher in reading and numeracy respectively than those without such problematic behaviour.

**Table 3:** Generalized linear model fitted to various scores and national standard scores for weekdays internet use adjusting the behavioral and sociodemographic factors, as well as survey weights.

Covariates	Reading score*		Writing score*		Numeracy score*		National standard score	
	OR (95% CI)	*p* value	OR (95% CI)	*p* value	OR (95% CI)	*p* value	OR (95% CI)	*p* value
Model 1**								
**Internet use (ref: ≤ 2 h)**								
2–4 h	0.971 (0.868, 1.086)	0.606	0.999 (0.894, 1.117)	0.989	0.916 (0.822, 1.02)	0.110	1.076 (0.78, 1.484)	0.657
> 4 h	0.852 (0.735, 0.987)	0.032	0.981 (0.856, 1.124)	0.783	0.831 (0.717, 0.964)	0.015	1.055 (0.714, 1.558)	0.790
Model 2***								
**Internet use (ref: ≤ 2 h)**								
2–4 h	0.968 (0.867, 1.082)	0.567	0.994 (0.891, 1.11)	0.918	0.911 (0.82, 1.013)	0.086	1.063 (0.767, 1.471)	0.715
> 4 h	0.856 (0.739, 0.99)	0.037	0.982 (0.858, 1.124)	0.797	0.835 (0.721, 0.967)	0.016	1.057 (0.711, 1.569)	0.785
Model 3****								
**Internet use (ref: ≤ 2 h)**								
2–4 h	0.948 (0.847, 1.06)	0.348	0.99 (0.884, 1.108)	0.858	0.896 (0.805, 0.997)	0.044	1.038 (0.748, 1.442)	0.822
> 4 h	0.822 (0.709, 0.953)	0.01	0.974 (0.849, 1.117)	0.709	0.809 (0.699, 0.936)	0.004	1.015 (0.683, 1.508)	0.943
Addiction tendency to internet or game-play	0.833 (0.728,0.954)	0.008	0.963 (0.841,1.102)	0.579	0.864 (0.755,0.987)	0.032	0.831 (0.558,1.239)	0.364

*Standardized (mean of 0 and a standard deviation of 1). **The model was adjusted for age and sex of the child, household income, primary carer’s education and family type. ***Model 2 was adjusted for age and sex of the child, household income, primary carer’s education, family type, remoteness, primary carer’s mental health, smoking and drinking habits. ****Model 3 was adjusted for age and sex of the child, household income, primary carer’s education, family type, remoteness, primary carer’s mental health, smoking and drinking habits and internet addiction of the child.

Internet use during weekends showed a positive association with academic performance (Table 4). For example, Model 1 in Table 4 shows that internet use during weekends was significant for reading, writing and national standard scores. Youths who spend around 2–4 h and over 4 h on the internet during weekends were 21% and 15% more likely to get a higher reading scores respectively compared to those who spend less than 2 h (Model 1, Table 4). Similarly, in model 3, where the internet addiction of adolescents was adjusted, adolescents who spent 2–4 h on internet were 1.59 times more likely to score above the national standard. All three models of Table 4 confirmed that adolescents who spent 2–4 h on the internet during weekends are more likely to achieve better reading and writing scores and be at or above national standard compared to those who used the internet for less than 2 h. Numeracy scores were unlikely to be affected by internet use. The results obtained from Model 3 should be treated as robust, as this is the most comprehensive model that accounts for unobserved characteristics. The addiction tendency to internet/game-play variable showed a negative association with academic performance, but this is only significant for numeracy scores.

**Table 4:** Generalized linear model fitted to various scores and national standard scores for weekends internet use adjusting the behavioural and sociodemographic factors, as well as survey weights.

	Reading score*		Writing score*		Numeracy score*		National standard score	
	OR (95% CI)	*p* value	OR (95% CI)	*p* value	OR (95% CI)	*p* value	OR (95% CI)	*p* value
Model 1**								
**Internet use (ref: ≤2h)**								
2–4 h	1.211 (1.071, 1.369)	0.002	1.237 (1.097,1.394)	0.001	1.094 (0.971,1.233)	0.139	1.677 (1.196,2.35)	0.003
> 4 h	1.15 (1.003, 1.319)	0.046	1.141 (0.998, 1.306)	0.054	0.971 (0.849, 1.112)	0.673	1.287 (0.888, 1.865)	0.183
Model 2***								
**Internet use (ref: ≤2h)**								
2–4 h	1.182 (1.046, 1.336)	0.007	1.205 (1.07, 1.356)	0.002	1.055 (0.938, 1.187)	0.372	1.61 (1.139, 2.275)	0.007
> 4 h	1.146 (1.00, 1.313)	0.05	1.129 (0.988, 1.29)	0.074	0.955 (0.836, 1.092)	0.504	1.281 (0.875, 1.876)	0.203
Model 3****								
**Internet use (ref: ≤2h)**								
2–4 h	1.171 (1.036,1.323)	0.011	1.204 (1.068,1.357)	0.002	1.045 (0.929,1.175)	0.468	1.591 (1.123,2.253)	0.009
> 4 h	1.102 (0.958,1.269)	0.174	1.124 (0.975,1.295)	0.106	0.917 (0.8,1.051)	0.213	1.223 (0.827,1.81)	0.313
Addiction tendency to internet or game-play	0.879 (0.765,1.009)	0.068	0.985 (0.857,1.133)	0.837	0.872 (0.760,1.002)	0.053	0.848 (0.566,1.27)	0.423

*Standardized (mean of 0 and a standard deviation of 1). **The model was adjusted for age and sex of the child, household income, primary carer’s education and family type. ***Model 2 was adjusted for age and sex of the child, household income, primary carer’s education, family type, remoteness, primary carer’s mental health, smoking and drinking habits. ****Model 3 was adjusted for age and sex of the child, household income, primary carer’s education, family type, remoteness, primary carer’s mental health, smoking and drinking habits and internet addiction of the child.

### Electronic gaming and academic performance

Time spent on electronic gaming during weekdays had no effect on the academic performance of writing and language but had significant association with reading scores (Model 2, Table 5). Model 2 of Table 5 shows that adolescents who spent 1–2 h on gaming during weekdays were 13% more likely to get higher reading scores compared to those who did not play at all. It was an interesting result that while electronic gaming during weekdays tended to show a positive effect on reading scores, internet use during weekdays showed a negative effect. Addiction tendency to internet/game-play had a negative effect; the adolescents who were addicted to the internet were 14% less likely to score more highly in reading than those without any such behaviour.

**Table 5:** Generalized linear model fitted to various scores and national standard scores for weekdays game-play adjusting the behavioural and sociodemographic factors, as well as survey weights.

Covariates	Reading score*		Writing score*		Numeracy score*		National standard score	
	OR (95% CI)	*p* value	OR (95% CI)	*p* value	OR (95% CI)	*p* value	OR (95% CI)	*p* value
Model 1**								
**Game-play (ref: No game/ 0 h)**								
1–2 h	1.121 (0.989,1.271)	0.075	1.089 (0.961,1.234)	0.181	1.074 (0.954,1.21)	0.239	1.188 (0.779,1.811)	0.423
> 2 h	1.017 (0.86,1.203)	0.843	0.957 (0.81,1.131)	0.607	0.872 (0.746,1.02)	0.086	0.753 (0.467,1.215)	0.245
Model 2***								
**Game-play (ref: No game/ 0 h)**								
1–2 h	1.132 (1,1.282)	0.05	1.084 (0.962,1.222)	0.184	1.103 (0.977,1.245)	0.113	1.234 (0.803,1.897)	0.337
> 2 h	1.044 (0.885,1.232)	0.609	0.891 (0.763,1.04)	0.144	0.985 (0.838,1.158)	0.857	0.803 (0.492,1.309)	0.379
Model 3****								
**Game-play (ref: No game/ 0 h)**								
1–2 h	1.124 (0.992,1.274)	0.066	1.081 (0.959,1.218)	0.201	1.097 (0.971,1.24)	0.138	1.218 (0.793,1.871)	0.367
> 2 h	1.011 (0.855,1.195)	0.897	0.878 (0.753,1.024)	0.099	0.961 (0.817,1.131)	0.632	0.757 (0.461,1.244)	0.273
Addiction tendency to internet or game-play	0.856 (0.748,0.979)	0.023	0.935 (0.820,1.065)	0.311	0.887 (0.774,1.015)	0.082	0.751 (0.497,1.135)	0.174

*Standardized (mean of 0 and a standard deviation of 1). **The model was adjusted for age and sex of the child, household income, primary carer’s education and family type. ***Model 2 was adjusted for age and sex of the child, household income, primary carer’s education, family type, remoteness, primary carer’s mental health, smoking and drinking habits. ****Model 3 was adjusted for age and sex of the child, household income, primary carer’s education, family type, remoteness, primary carer’s mental health, smoking and drinking habits and internet addiction of the child.

All three models from Table 6 confirm that time spent on electronic gaming over 2 h during weekends had a positive effect on readings scores. For example, the results of Model 3 (Table 6) showed that adolescents who spent more than 2 h on electronic gaming during weekdays were 16% more likely to have better reading scores compared to adolescents who did not play games at all. Playing electronic games during weekends was not found to be statistically significant for writing and numeracy scores and national standard scores, although the odds ratios were positive. The results from all tables confirm that addiction tendency to internet/gaming is negatively associated with academic performance, although the variable is not always statistically significant.

**Table 6:** Generalized linear model fitted to various scores and national standard scores for weekends game-play adjusting the behavioural and sociodemographic factors, as well as survey weights.

	Reading score*		Writing score*		Numeracy score*		National standard score	
	OR (95% CI)	*p* value	OR (95% CI)	*p* value	OR (95% CI)	*p* value	OR (95% CI)	*p* value
Model 1**								
**Game-play (ref: No game/ 0 h)**								
1–2 h	1.064 (0.935,1.212)	0.344	1.045 (0.925,1.181)	0.479	1.058 (0.93,1.202)	0.392	1.127 (0.738,1.722)	0.579
> 2 h	1.172 (1.01,1.359)	0.036	1.001 (0.869,1.153)	0.985	1.068 (0.92,1.239)	0.386	0.953 (0.597,1.522)	0.841
Model 2***								
**Game-play (ref: No game/ 0 h)**								
1–2 h	1.08 (0.95,1.228)	0.241	1.059 (0.937,1.197)	0.361	1.077 (0.951,1.22)	0.245	1.179 (0.765,1.816)	0.455
> 2 h	1.186 (1.025,1.372)	0.022	1.01 (0.878,1.162)	0.885	1.082 (0.936,1.249)	0.287	0.998 (0.619,1.608)	0.992
Model 3****								
**Game-play (ref: No game/ 0 h)**								
1–2 h	1.077 (0.946,1.224)	0.262	1.058 (0.936,1.196)	0.369	1.074 (0.948,1.217)	0.263	1.172 (0.761,1.806)	0.471
> 2 h	1.16 (1.002,1.343)	0.047	1.003 (0.872,1.153)	0.969	1.062 (0.918,1.229)	0.417	0.957 (0.592,1.548)	0.859
Addiction tendency to internet or game-play	0.887 (0.776,1.013)	0.077	0.96 (0.841,1.095)	0.540	0.906 (0.791,1.038)	0.154	0.793 (0.529,1.187)	0.260

*Standardized (mean of 0 and a standard deviation of 1). **The model was adjusted for age and sex of the child, household income, primary carer’s education and family type. ***Model 2 was adjusted for age and sex of the child, household income, primary carer’s education, family type, remoteness, primary carer’s mental health, smoking and drinking habits. ****Model 3 was adjusted for age and sex of the child, household income, primary carer’s education, family type, remoteness, primary carer’s mental health, smoking and drinking habits and internet addiction of the child.

## Discussion

Building on past research on the effect of the internet use and electronic gaming in adolescents, this study examined whether Internet use and playing electronic games were associated with academic performance (i.e. reading, writing and numeracy) using a standardized test of academic performance (i.e. NAPLAN) in a nationally representative dataset in Australia. The findings of this study question the conventional belief^9,25^ that academic performance is negatively associated with internet use and electronic games, particularly when the internet is used for non-academic purpose.

In the current hi-tech world, many developed countries (e.g. the USA, Canada and Australia) have recommended that 5–17 year-olds limit electronic media (e.g. internet, electronic games) to 2 h per day for entertainment purposes, with concerns about the possible negative consequences of excessive use of electronic media^14,26^. However, previous research has often reported that children and adolescents spent more than the recommended time^26^. The present study also found similar results, that is, that about 70% of the sampled adolescents aged 11–17 spent more than 2 h per day on the Internet and nearly 30% spent more than 2-h on electronic gaming in a day. This could be attributed to the increased availability of computers/smart-phones and the internet among under-18s^12^. For instance, 97% of Australian households with children aged less than 15 years accessed internet at home in 2016–2017^10^; as a result, policymakers recommended that parents restrict access to screens (e.g. Internet and electronic games) in children’s bedrooms, monitor children using screens, share screen hours with their children, and to act as role models by reducing their own screen time^14^.

This research has drawn attention to the fact that the average time spent using the internet, which is often more than 4 h during weekdays tends to be negatively associated with academic performance, especially a lower reading and numeracy score, while internet use of more than 2 h during weekends is positively associated with academic performance, particularly having a better reading and writing score and above national standard score. By dividing internet use and gaming by weekdays and weekends, this study find an answer to the mixed evidence found in previous literature^9^. The results of this study clearly show that the non-academic use of internet during weekdays, particularly, spending more than 4 h on internet is harmful for academic performance, whereas, internet use on the weekends is likely to incur a positive effect on academic performance. This result is consistent with a USA study that reported that internet use is positively associated with improved reading skills and higher scores on standardized tests^13,27^. It is also reported in the literature that academic performance is better among moderate users of the internet compared to non-users or high level users^13,27^, which was in line with the findings of this study. This may be due to the fact that the internet is predominantly a text-based format in which the internet users need to type and read to access most websites effectively^13^. The results of this study indicated that internet use is not harmful to academic performance if it is used moderately, especially, if ensuring very limited use on weekdays. The results of this study further confirmed that timing (weekdays or weekends) of internet use is a factor that needs to be considered.

Regarding electronic gaming, interestingly, the study found that the average time of gaming either in weekdays or weekends is positively associated with academic performance especially for reading scores. These results contradicted previous literatures^1,13,19,27^ that have reported negative correlation between electronic games and educational performance in high-school children. The results of this study were consistent with studies conducted in the USA, Europe and other countries that claimed a positive correlation between gaming and academic performance, especially in numeracy and reading skills^28,29^. This is may be due to the fact that the instructions for playing most of the electronic games are text-heavy and many electronic games require gamers to solve puzzles^9,30^. The literature also found that playing electronic games develops cognitive skills (e.g. mental rotation abilities, dexterity), which can be attributable to better academic achievement^31,32^.

Consistent with previous research findings^33–36^, the study also found that adolescents who had addiction tendency to internet usage and/or electronic gaming were less likely to achieve higher scores in reading and numeracy compared to those who had not problematic behaviour. Addiction tendency to Internet/gaming among adolescents was found to be negatively associated with overall academic performance compared to those who were not having addiction tendency, although the variables were not always statistically significant. This is mainly because adolescents’ skipped school and missed classes and tuitions, and provide less effort to do homework due to addictive internet usage and electronic gaming^19,35^. The results of this study indicated that parental monitoring and/ or self-regulation (by the users) regarding the timing and intensity of internet use/gaming are essential to outweigh any negative effect of internet use and gaming on academic performance.

Although the present study uses a large nationally representative sample and advances prior research on the academic performance among adolescents who reported using the internet and playing electronic games, the findings of this study also have some limitations that need to be addressed. Firstly, adolescents who reported on the internet use and electronic games relied on self-reported child data without any screening tests or any external validation and thus, results may be overestimated or underestimated. Second, the study primarily addresses the internet use and electronic games as distinct behaviours, as the YMM survey gathered information only on the amount of time spent on internet use and electronic gaming, and included only a few questions related to addiction due to resources and time constraints and did not provide enough information to medically diagnose internet/gaming addiction. Finally, the cross-sectional research design of the data outlawed evaluation of causality and temporality of the observed association of internet use and electronic gaming with the academic performance in adolescents.

## Conclusion

This study found that the average time spent on the internet on weekends and electronic gaming (both in weekdays and weekends) is positively associated with academic performance (measured by NAPLAN) of Australian adolescents. However, it confirmed a negative association between addiction tendency (internet use or electronic gaming) and academic performance; nonetheless, most of the adolescents used the internet and played electronic games more than the recommended 2-h limit per day. The study also revealed that further research is required on the development and implementation of interventions aimed at improving parental monitoring and fostering users’ self-regulation to restrict the daily usage of the internet and/or electronic games.

## Methods

### Data description

Young minds matter (YMM) was an Australian nationwide cross-sectional survey, on children aged 4–17 years conducted in 2013–2014^37^. Out of the initial 76,606 households approached, a total of 6,310 parents/caregivers (eligible household response rate 55%) of 4–17 year-old children completed a structured questionnaire via face to face interview and 2967 children aged 11–17 years (eligible children response rate 89%) completed a computer-based self-reported questionnaire privately at home^37^.

Area based sampling was used for the survey. A total of 225 Statistical Area 1 (defined by Australian Bureau of Statistics) areas were selected based on the 2011 Census of Population and Housing. They were stratified by state/territory and by metropolitan versus non-metropolitan (rural/regional) to ensure proportional representation of geographic areas across Australia^38^. However, a small number of samples were excluded, based on most remote areas, homeless children, institutional care and children living in households where interviews could not be conducted in English. The details of the survey and methodology used in the survey can be found in Lawrence et al.^37^.

Following informed consent (both written and verbal) from the primary carers (parents/caregivers), information on the National Assessment Program—Literacy and Numeracy (NAPLAN) of the children and adolescents were also added to the YMM dataset. The YMM survey is ethically approved by the Human Research Ethics Committee of the University of Western Australia and by the Australian Government Department of Health. In addition, the authors of this study obtained a written approval from Australian Data Archive (ADA) Dataverse to access the YMM dataset. All the researches were done in accordance with relevant ADA Dataverse guidelines and policy/regulations in using YMM datasets.

### Variables

#### Outcome variables

The NAPLAN, conducted annually since 2008, is a nationwide standardized test of academic performance for all Australian students in Years 3, 5, 7 and 9 to assess their skills in reading, writing numeracy, grammar and spelling^39,40^. NAPLAN scores from 2010 to 2015, reported by YMM, were used as outcome variables in the models; while NAPLAN data of 2008 (N = 4) and 2009 (N = 29) were excluded for this study in order to reduce the time lag between YMM survey and the NAPLAN test. The NAPLAN gives point-in-time standardized scores, which provide the scope to compare children’s academic performance over time^40,41^. The NAPLAN tests are one component of the evaluation and grading phase of each school, and do not substitute for the comprehensive, consistent evaluations provided by teachers on the performance of each student^39,41^. All four domains—reading, writing, numeracy and language conventions (grammar and spelling) are in continuous scales in the dataset. The scores are given based on a series of tests; details can be found in^42^. The current study uses only reading, writing and numeracy scores to measure academic performance.

In this study, the National standard score is a combination of three variables: whether the student meets the national standard in reading, writing and numeracy. Based on national average score, a binary outcome variable is also generated. One category is ‘below standard’ if a child scores at least one standard deviation (one below scores) from the national standard in reading, writing and numeracy, and the rest is ‘at/above standard’.

### Independent variables

#### Internet use and electronic gaming

In the YMM survey, owing to the scope of the survey itself, an extensive set of questions about internet usage and electronic gaming could not be included. Internet usage omitted the time spent in academic purposes and/or related activities. Playing electronic games included playing games on a gaming console (e.g. PlayStation, Xbox, or similar console ) online or using a computer, or mobile phone, or a handled device^12^. The primary independent covariates were average internet use per day and average electronic game-play in hours per day. A combination of hours on weekdays and weekends was separately used in the models. These variables were based on a self-assessed questionnaire where the youths were asked questions regarding daily time spent on the Internet and electronic game-play, specifically on either weekends or weekdays. Since, internet use/game-play for a maximum of 2 h/day is recommended for children and adolescents aged between 5 and 17 years in many developed countries including Australia^14,26^; therefore, to be consistent with the recommended time we preferred to categorize both the time variables of internet use and gaming into three groups with an interval of 2 h each. Internet use was categorized into three groups: (a) ≤ 2 h), (b) 2–4 h, and (c) > 4 h. Similar questions were asked for game-play h. The sample distribution for electronic game-play was skewed; therefore, this variable was categorized into three groups: (a) no game-play (0 h), (b) 1–2 h, and (c) > 2 h.

### Other covariates

Family structure and several sociodemographic variables were used in the models to adjust for the differences in individual characteristics, parental inputs and tastes, household characteristics and place of residence. Individual characteristics included age (continuous) and sex of the child (boys, girls) and addiction tendency to internet use and/or game-play of the adolescent. Addiction tendency to internet/game-play was a binary independent variable. It was a combination of five behavioural questions relating to: whether the respondent avoided eating/sleeping due to internet use or game-play; feels bothered when s/he cannot access internet or play electronic games; keeps using internet or playing electronic games even when s/he is not really interested; spends less time with family/friends or on school works due to internet use or game-play; and unsuccessfully tries to spend less time on the internet or playing electronic games. There were four options for each question: never/almost never; not very often; fairly often; and very often. A binary covariate was simulated, where if any four out of five behaviours were reported as for example, fairly often or very often, then it was considered that the respondent had addictive tendency.

Household characteristics included household income (low, medium, high), family type (original, step, blended, sole parent/primary carer, other)^43^ and remoteness (major cities, inner regional, outer regional, remote/very remote). Parental inputs and taste included education of primary carer (bachelor, diploma, year 10/11), primary carer’s likelihood of serious mental illness (K6 score -likely; not likely); primary carer’s smoking status (no, yes); and risk of alcoholic related harm by the primary carer (risky, none).

### Statistical analysis

Descriptive statistics of the sample and distributions of the outcome variables were initially assessed. Based on these distributions, the categorization of outcome variables was conducted, as mentioned above. For formal analysis, generalized linear regression models (GLMs)^44^ were used, adjusting for the survey weights, which allowed for generalization of the findings. As NAPLAN scores of three areas—reading, writing and numeracy—were continuous variables, linear models were fitted to daily average internet time and electronic game play time. The scores were standardized (mean = 0, SD = 1) for model fitness. The binary logistic model was fitted for the dichotomized national standard outcome variable. Separate models were estimated for internet and electronic gaming on weekends and weekdays.

We estimated three different models, where models varied based on covariates used to adjust the GLMs. Model 1 was adjusted for common sociodemographic factors including age and sex of the child, household income, education of primary carer’s and family type^43^. However, the results of this model did not account for some unobserved household characteristics (e.g. taste, preferences) that are unobserved to the researcher and are arguably correlated with potential outcomes. The effects of unobserved characteristics were reduced by using a comprehensive set of observable characteristics^45,46^ that were available in YMM data. The issue of unobserved characteristics was addressed by estimating two additional models that include variables by including household characteristics such as parental taste, preference and inputs, and child characteristics in the model. In addition to the variables in Model 1, Model 2 included remoteness, primary carer’s mental health status, smoking status and risk of alcoholic related harm by the primary carer. Model 3 further included internet/game addiction of the adolescent in addition to all the covariates in Model 2. Model 3 was expected to account for a child’s level of unobserved characteristics as the children who were addicted to internet/games were different from others. The model will further show how academic performance is affected by internet/game addiction. The correlation among the variables ‘internet/game addiction’ and ‘internet use’ and ‘gaming’ (during weekdays and weekends) were also assessed, and they were less than 0.5. Multicollinearity was assessed using the variance inflation factor (VIF), which was under 5 for all models, suggesting no multicollinearity^47^.

*p* value below the threshold of 0.05 was considered the threshold of significance. All analysis was conducted in *R* (version 3.6.1). R-package *survey* (version 3.37) was used for modelling which is suited for complex survey samples^48^.

## Data Availability

The authors declare that they do not have permission to share dataset. However, the datasets of Young Minds Matter (YMM) survey data is available at the Australian Data Archive (ADA) Dataverse on request (https://doi.org/10.4225/87/LCVEU3).
